# Plasmablastic non-Hodgkin’s lymphoma: a case report and review of the literature

**DOI:** 10.1186/s13256-026-06417-w

**Published:** 2026-07-29

**Authors:** Imran Saleem Solanki, Rajanikanth Kambala, Nitin Bhola, Sanjana Wadeawale

**Affiliations:** Department of Oral and Maxillofacial Surgery, Sharad Pawar Dental College and Hospital, Datta Meghe Institute of Higher Education and Research, Sawangi, Wardha, India

**Keywords:** Plasmablastic lymphoma (PBL), Non-Hodgkin lymphoma, HIV-associated lymphoma, Oral cavity lymphoma, Immunohistochemistry, Ki-67 proliferative index

## Abstract

**Background:**

Plasmablastic lymphoma (PBL) is an unusual and aggressive form of non-Hodgkin lymphoma that most frequently occurs in immunosuppressed patients, especially those suffering from HIV infection. The association between PBL and the oral cavity is very rare, and the clinical features may mimic odontogenic and epithelial tumors, which causes a delayed diagnosis. Here, we describe an extremely rare case of localized PBL involving the anterior mandible in an HIV-infected patient clinically resembling carcinoma of the lower anterior alveolus.

**Case presentation:**

A 43-year-old man, HIV positive, Indian origin, with progressive enlargement of painful swelling over chin area for three months was seen. Examination showed a hard swelling with hypopigmented skin over the lower anterior chin area. Intraorally, there was presence of a nodular, erythematous, pebble like appearance of the mucosa in the edentulous anterior mandibular region, without any ulcerations. No cervical lymphadenopathy could be palpated. Clinically, the provisional diagnosis was made as carcinoma of the lower anterior alveolus, with lymphoma and Kaposi sarcoma being the differentials. Contrast-enhanced computed tomography scan showed a destructive mass with destruction of the anterior mandible and involvement of the surrounding soft tissue areas. Histopathology showed diffuse sheets of plasmablast-like cells. Immunohistochemistry showed MUM-1 and CD45 positivity and CD20 and PAX-5 negativity and high ki67 proliferation index (80–85%). Thus, the diagnosis of plasmablastic lymphoma was confirmed. The staging showed Stage IE extranodal disease without any significant lymphadenopathy and systemic symptoms. The patient was put on antiretroviral and systemic chemotherapeutic treatment.

**Conclusions:**

This case demonstrates the difficulty in making a diagnosis for plasmablastic lymphoma mimicking a localized mandibular mass, similar to an oral squamous cell carcinoma. The differential diagnosis must include lymphoma in cases where there is an unusual mandibular swelling, especially when there is an underlying immunocompromised status. Imaging, histopathologic, and immunohistologic studies are crucial in making an early diagnosis and appropriate management of the disease. This case report illustrates the significance of a multidisciplinary management strategy for an unusual oral malignancy.

## Background

Plasmablastic lymphoma (PBL) is a rare, highly aggressive subtype of diffuse large B-cell lymphoma that was first described by Delecluse*et al*. in 1997 as an oral cavity-associated lymphoma in patients with human immunodeficiency virus (HIV) infection. Although initially recognized as an HIV-associated malignancy, subsequent reports have documented its occurrence in HIV-negative and other immunocompromised individuals. PBL accounts for a small proportion of all non-Hodgkin lymphomas but is associated with rapid disease progression, a high proliferative index, and poor overall survival. Histologically, it is characterized by plasmablastic morphology with loss of conventional B-cell markers such as CD20 and PAX5 and expression of plasma cell-associated markers including CD138, CD38, and MUM1, making immunohistochemistry indispensable for establishing the diagnosis and differentiating it from plasmablastic myeloma and other high-grade lymphomas. The oral cavity is the most frequently affected extranodal site, particularly the gingiva, palate, and jaws; however, primary involvement of the anterior mandible is distinctly uncommon. Because the clinical and radiographic features often resemble oral squamous cell carcinoma, odontogenic malignancies, chronic osteomyelitis, or severe periodontal disease, diagnosis is frequently delayed until histopathological and immunohistochemical evaluation is performed. Current treatment generally consists of intensive multi-agent chemotherapy, with highly active antiretroviral therapy serving as an essential adjunct in HIV-positive patients, although the prognosis remains guarded owing to the tumor's aggressive biological behavior and high risk of systemic dissemination. Reports of localized anterior mandibular PBL presenting as a carcinoma-like lesion are limited, highlighting the need for increased awareness among oral and maxillofacial surgeons, oral pathologists, and oncologists. The aim of this case report is to describe a rare case of Stage IE plasmablastic lymphoma involving the anterior mandible in an HIV-positive patient that clinically mimicked carcinoma of the lower anterior alveolus, emphasizing the diagnostic challenges, the pivotal role of histopathological and immunohistochemical evaluation in achieving an accurate diagnosis, and the importance of early multidisciplinary management to facilitate prompt treatment and improve clinical outcomes.

## Case report

**Fig. 1:**
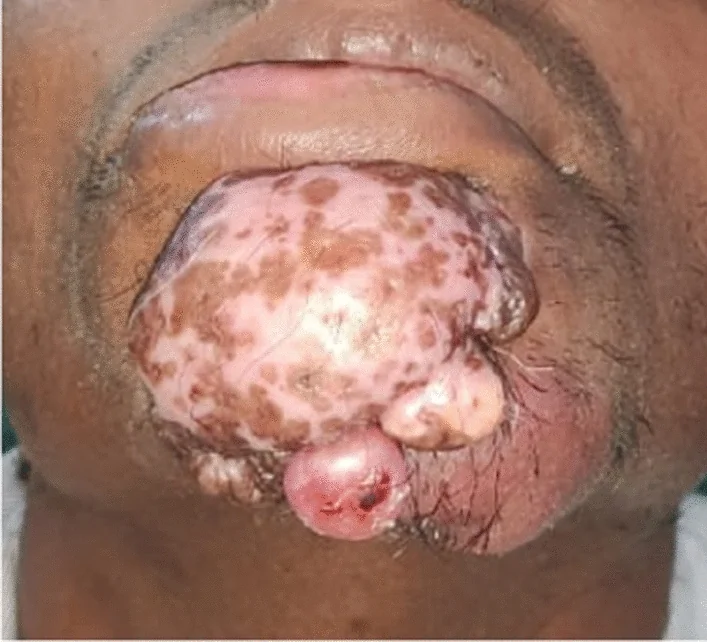
Extraoral clinical photograph

A 43‑year‑old Indian male presented to the outpatient department of Oral & Maxillofacial Surgery with Painful swelling over the chin region that had been gradually increasing in size over past three months (Fig. 1). The was patient recently diagnosed with HIV-positive and was started on regular antiretroviral therapy (ART). He denied any history of fever or weight loss.

**Fig. 2:**
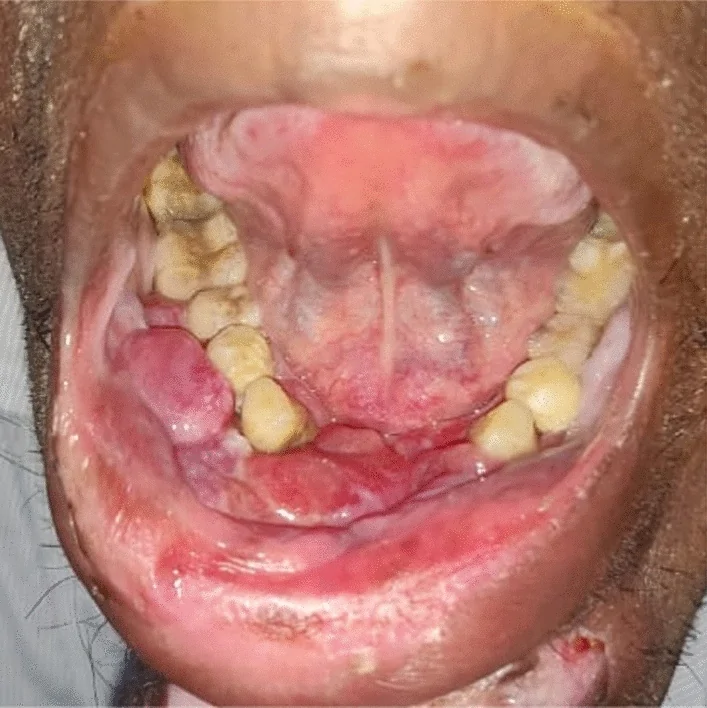
Intraoral view demonstrating nodular, erythematous, and pebbly mucosal changes

On clinical examination, extraoral examination (Fig. 1) revealed a firm, non-tender swelling approximately 3.5 × 2.5 cm over the midline of the chin, fixed to the underlying bone and involving the overlying skin with hypopigmentation. Clinically cervical lymph nodes are non-palpable. Intraorally (Fig. 2) there was partial edentulism in the anterior mandible. The mucosa appeared nodular, erythematous, and pebbly with no ulceration or discharge. On palpation consistency was firm, induration and tenderness were present. Based on the history and clinical findings, a provisional diagnosis of carcinoma of lower anterior alveolus with a differential diagnosis lymphoma and Kaposi sarcoma was made.

**Fig. 3:**
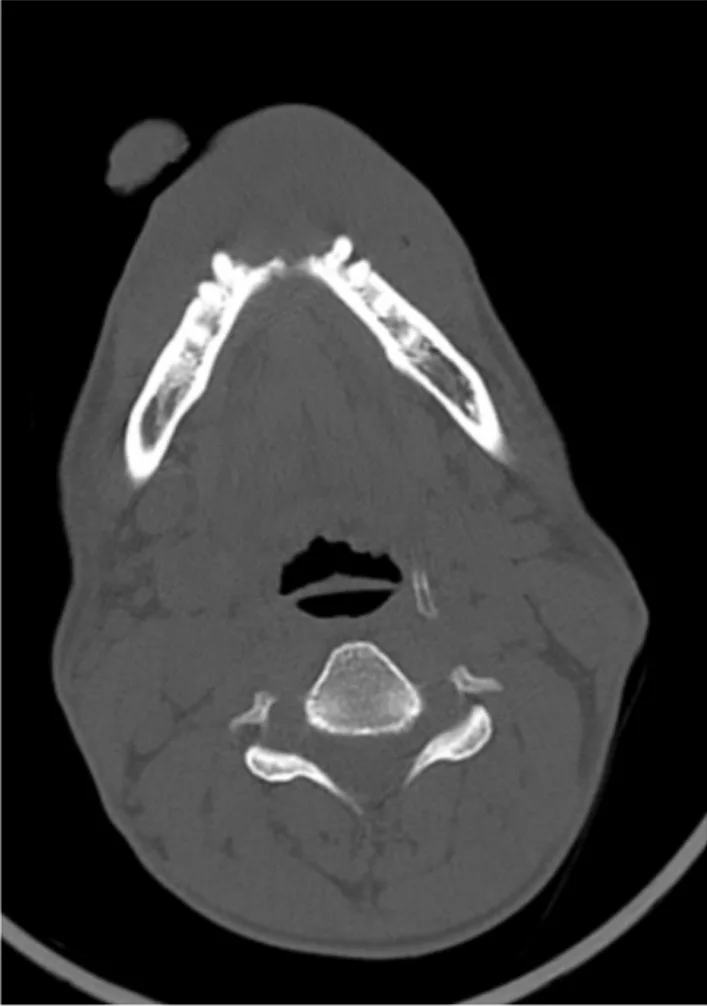
Axial bony computed tomography image showing erosion of the mandibular body in the anterior (central arch) region

**Fig. 4:**
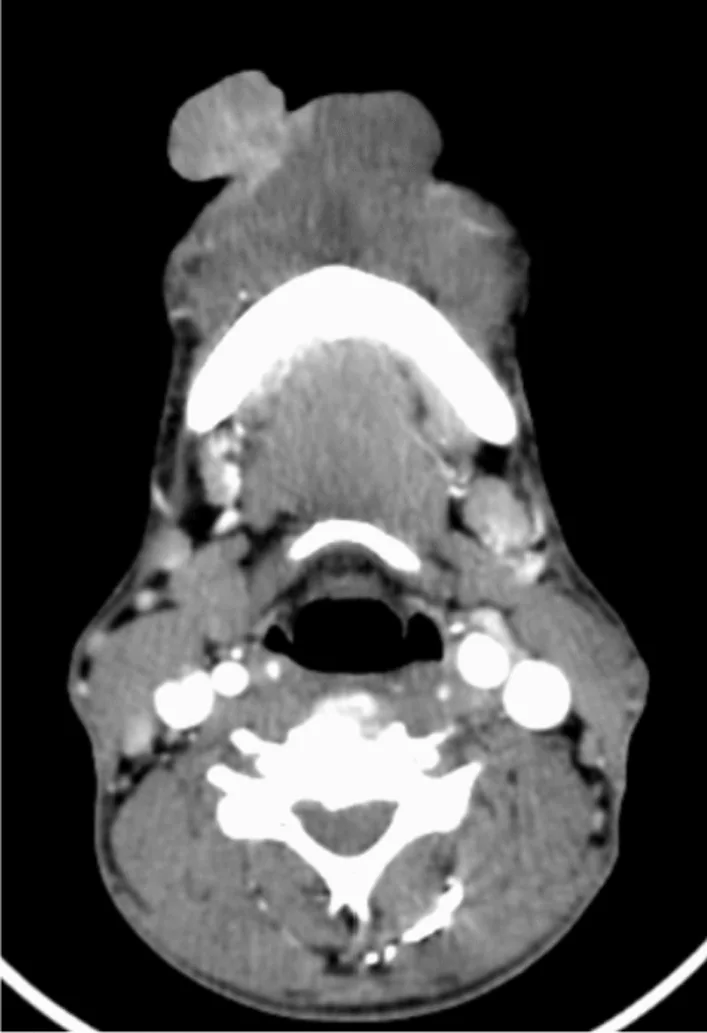
Axial soft tissue computed tomography image revealing a heterogeneously enhancing soft tissue mass

A radiological, histopathological and routine hematological investigations was performed. The contrast enhanced computed tomography (CECT) of head and neck revealed irregular proliferative heterogeneously enhancing soft tissue density mass lesion noted. Posteriorly lesion causes erosion of mandibular body (Central arch) partially invades floor of mouth. Bony erosion can be appreciated in Fig. 3 and the soft tissue involvement and the fungation in Fig. 4. CECT thorax and abdomen revealed no abnormality.

**Fig. 5:**
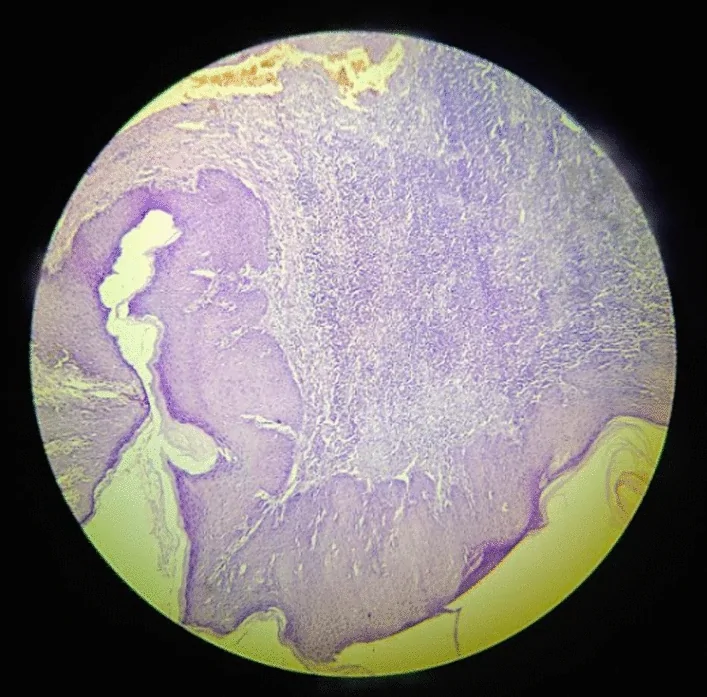
Histopathological photomicrograph suggestive of plasmablastic lymphoma (hematoxylin and eosin stain)

**Fig. 6:**
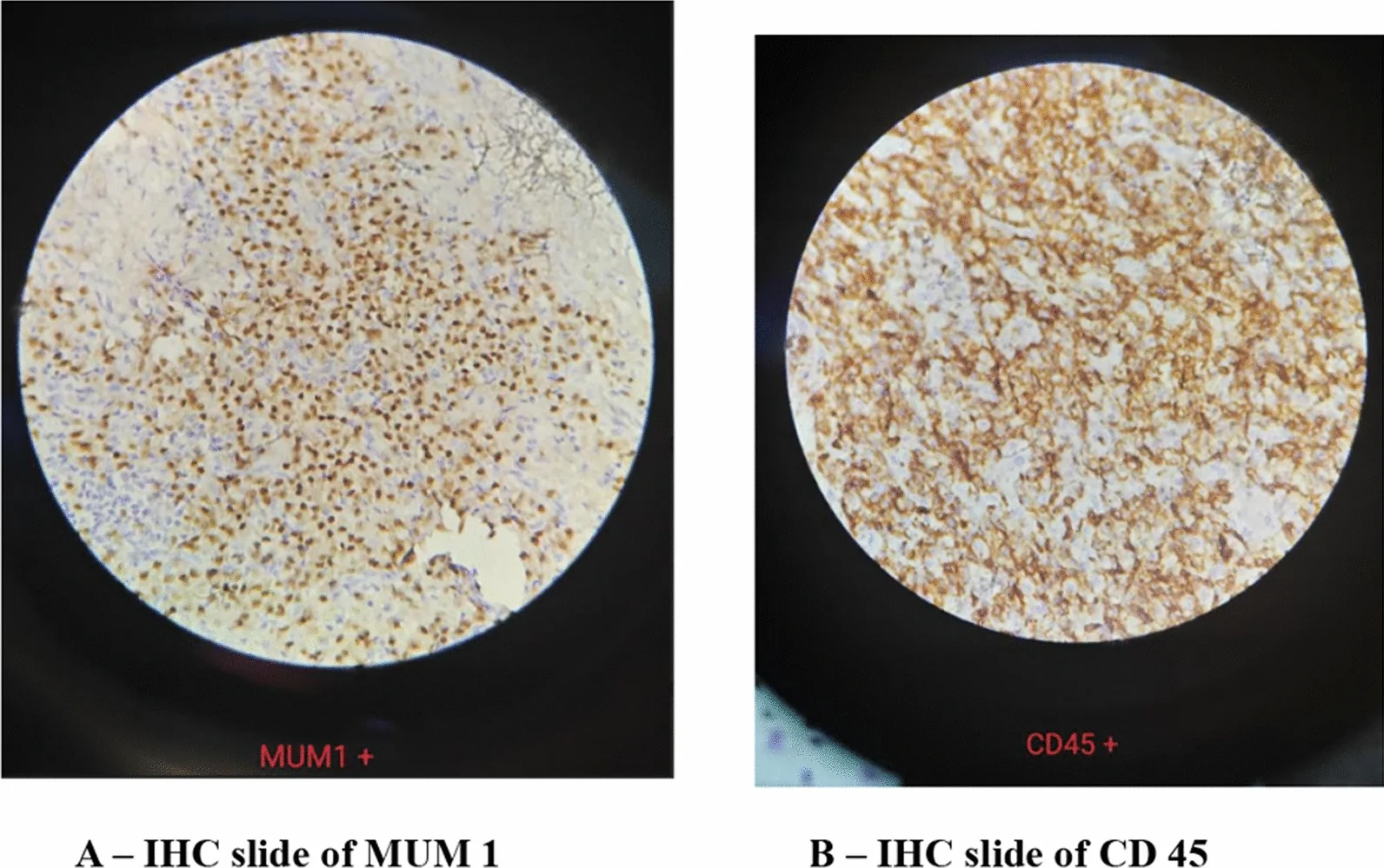
**A** Immunohistochemical staining showing tumor cells positive for MUM-1.** B** Immunohistochemical staining showing tumor cells positive for CD45

Biopsy revealed normal looking stratified squamous epithelium. Underlying dermis showed monomorphous intermediate to large round cell with hyperchromatic, eccentrically placed nucleus and prominent nucleoli with basophilic cytoplasm-plasmablast like cells. Impression of the histopath report—histopathology features are of small round cell tumor—non-Hodgkin’s lymphoma (mostly plasmablastic type)—Fig. 5. Immunohistochemistry (IHC) was performed in which the tumor cells were positive for MUM-1 (Fig. 6A) and CD-45 (Fig. 6B) while negative for CD-20 and PX-5 was reported.

**Fig. 7:**
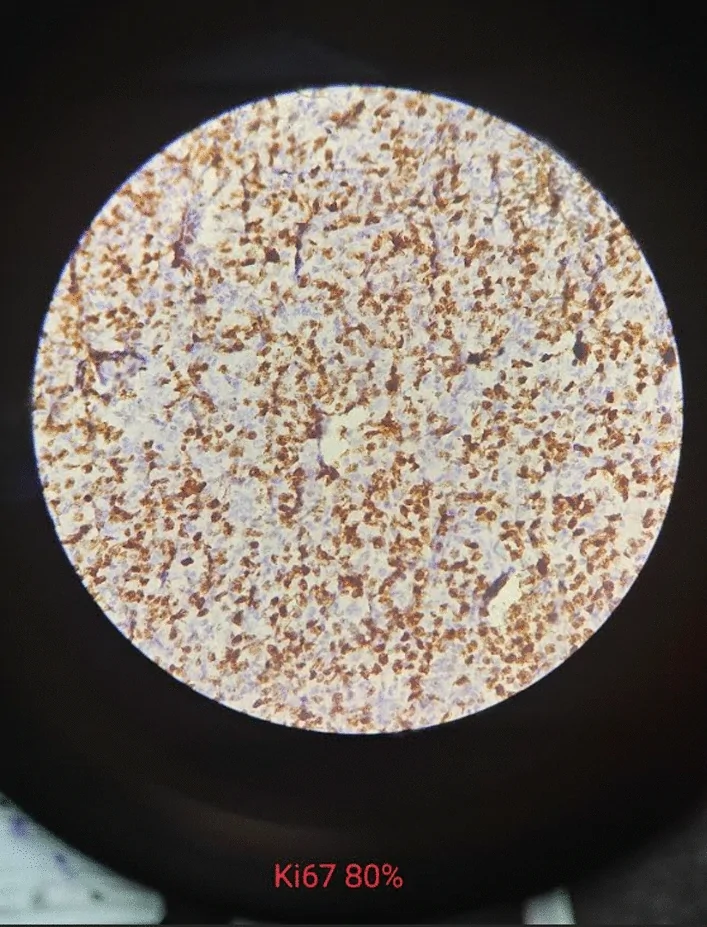
Immunohistochemical staining demonstrating a high Ki-67 proliferative index (approximately 80–85%), indicating high tumor proliferation

The Ki-67 proliferative index is high 80–85% (Fig. 7) impression—the histopathological and immunohistochemistry findings are suggestive of plasmablastic lymphoma.

A whole-body scan revealed localized disease without nodal or distant involvement, diagnosis of Stage IE primary extranodal lymphoma was made. The treatment was planned chemotherapy through R-CHOP regimen (rituximab, cyclophosphamide, doxorubicin, vincristine, and prednisone (6 cycles). ART with tenofovir, lamivudine, and efavirenz to be continued.

## Discussion

Extranodal non-Hodgkin’s lymphomas (NHLs) are lymphoid malignancies that arise outside the lymph nodes and account for about 20–30% of all NHLs. Among extranodal sites, involvement of the head and neck region is relatively rare, although it is the second most common site after the gastrointestinal tract [1].

Primary lymphomas in the head and neck area account for 2.2% of all head neck cancers and 3.5% of intraoral cancers, making them the second or third most prevalent malignancies behind squamous cell carcinomas and salivary gland tumors. Three main types of lymphoid malignancies are recognized by the World Health Organization’s (WHO's) revision of the Revised EuropeanAmerican Lymphoma Classification: Hodgkin's lymphoma, T-cell/NK cell lymphoma, and B-cell lymphoma [2].

Plasmablastic lymphoma (PBL) is an uncommon but aggressive subtype of diffuse, large Bcell lymphoma. Despite significant advances in the field of lymphomas since this subtype was first described in a case series by Delecluse *et al* in 1997, PBL continues to pose challenges with diagnosis and management. [3].

It is classically seen in association with HIV infection. The primary organs involved are the gastrointestinal system, lymph nodes, oral mucosa, and sometimes the skin. PBL is diagnosed primarily through histologic evaluation with or without genetics and immunophenotyping. However, diagnosing PBL is difficult due to overlapping features with plasmablastic myeloma and other high-grade lymphomas with plasmablastic morphology [4].

Immunophenotyping can aid in diagnosing PBL as staining can help differentiate this condition from other plasma cell neoplasms. The immunophenotype for plasmablastic lymphoma is also similar to that of plasma cell neoplasms with positive immunohistochemical (IHC) staining for CD79a, IRF-4/MUM-1, CD38, and CD138. Unlike other B-cell lymphomas, PBL is usually negative for CD19, CD20, and PAX-5 but may be weakly positive for CD45 [5].

The Ki-67 proliferation index is invariably high and often exceeds 90% [6].

Immunohistochemistry showed differences between HIV positive and negative patients, the former had significantly higher CD20 and CD56 expression than the latter [7].

The high association of PBL with immunosuppression, oral cavity involvement, male gender, Epstein Barr Virus (EBV) infection, and aggressive clinical behavior has been traditionally stressed in the literature [8].

### Clinical mimicry of periodontal or infectious conditions

Patients with underlying neoplastic conditions affecting the oral cavity may initially present with signs and symptoms that closely mimic common periodontal or infectious diseases. Tooth mobility, localized pain, and swelling are frequent complaints that can be indistinguishable from advanced periodontitis or dental abscesses. Gingival enlargement or ulcerated lesions may resemble inflammatory hyperplasia or necrotizing ulcerative gingivitis, particularly in immunocompromised individuals such as those with HIV. Radiographic evaluation often reveals bone loss or osteolytic lesions, findings that can be misinterpreted as osteomyelitis or periodontal bone destruction. A crucial clinical indicator is the lack of response to conventional periodontal therapy or antibiotics; this should prompt clinicians to consider alternative diagnoses, including malignancies. In some cases, systemic symptoms like fever, unexplained weight loss, and generalized malaise accompany local oral findings, further complicating the clinical picture and potentially leading to an initial misdiagnosis of systemic infection rather than a neoplastic process [9].

This is a case of Stage IE primary extranodal plasmablastic lymphoma (PBL) involving the anterior mandible in an HIV-positive individual, clinically mimicking carcinoma of the lower anterior alveolus. The diagnosis was established based on imaging, histopathology, and immunohistochemistry findings.

### Clinical signs and symptoms in this case (consistent with NHL presentation)

**Table:** 

Category	Findings in this case	Typical in NHL
Local swelling	Painful, firm swelling over the chin (3.5 × 2.5 cm)	Common in extranodal NHL especially in oral cavity
Skin involvement	Hypopigmentation, lesion fixed to overlying skin	Seen in aggressive NHL variants like PBL
Oral findings	Nodular, erythematous mucosa without ulceration	Frequently seen in PBL of the oral cavity
Bone involvement	Mandibular body erosion, floor of mouth invasion on CECT	Bony invasion is common in oral NHL
Pain	Present	May be present, though not always
Tenderness	Present	Can occur with rapid tumor expansion
Systemic symptoms	Absent (no fever, no weight loss)	“B-symptoms” not always present in early-stage NHL
Lymphadenopathy	Not palpable	May be absent in localized extranodal disease
Histopathology	Small round blue cells, plasmablast-like morphology	Consistent with PBL features
Immunohistochemistry	Positive: MUM-1, CD45; negative: CD20, PX-5; high Ki-67 (80–85%)	Diagnostic hallmark of PBL

National Comprehensive Cancer Network (NCCN) guideline-informed approach for plasmablastic lymphoma (PBL), based on the NCCN’s B-Cell and HIV-related B-cell lymphomas recognized by NCCN under HIV-related B-cell lymphomas, often treated as a variant of diffuse large B-cell lymphoma (DLBCL) systemic chemotherapy

- R-CHOP is suboptimal: PBL is mostly CD20—so rituximab adds little value
- Preferred regimens recommended include:
  - Dose-adjusted EPOCH (etoposide, prednisone, vincristine, cyclophosphamide, doxorubicin)—highest-level NCCN-supported choice.
  - Hyper-Cyclophosphamide, Vincristine, Doxorubicin (Adriamycin), and Dexamethasone / Methotrexate (CVAD/MA) or Cyclophosphamide, Vincristine (Oncovin), Doxorubicin (Adriamycin), High-dose Methotrexate / Ifosfamide, Etoposide (VP-16), and High-dose Cytarabine (Ara-C) (CODOX-M/IVAC) for patients fit enough for intensive regimens 3. Central Nervous System (CNS) prophylaxis.
- Intrathecal methotrexate (± cytarabine) is advised for oral/jaw involvement due to elevated CNS relapse risk.

### Antiretroviral therapy (ART)

For HIV-positive patients, maintaining or initiating effective ART is essential to support treatment efficacy and immune recovery—consistent with NCCN policy for HIV-related lymphomas.

### Localized disease management

For Stage I–II disease, involved-site radiotherapy (ISRT) may be employed after chemo to improve local control [10].

## Conclusion

Plasmablastic lymphoma of the oral cavity is a rare but aggressive malignancy that can clinically mimic carcinoma or periodontal pathology. This case underscores the importance of considering lymphoma in the differential diagnosis of persistent oral swellings, particularly in immunocompromised patients. A multidisciplinary approach incorporating imaging, histopathology, immunohistochemistry, systemic chemotherapy, and continuation of antiretroviral therapy is essential for accurate diagnosis and effective management. Early detection and tailored treatment are vital to improving prognosis in this challenging disease entity.

### Main take-away lessons from this case report

- Plasmablastic lymphoma (PBL) is a rare, aggressive extranodal lymphoma that frequently involves the oral cavity, especially in HIV-positive patients.
- Oral PBL can closely mimic squamous cell carcinoma, periodontal disease, or infectious conditions, leading to diagnostic delays.
- Clinical red flags include a firm, destructive swelling with poor response to conventional periodontal or antibiotic therapy.
- Histopathology combined with immunohistochemistry (positive for MUM-1, CD45; negative for CD20, PAX-5; high Ki-67 index) is critical for accurate diagnosis.
- Imaging assists in staging, but tissue diagnosis is mandatory.
- Standard R-CHOP therapy may be suboptimal since PBL is usually CD20-negative; more intensive regimens such as dose-adjusted EPOCH may be preferable, along with antiretroviral therapy in HIV patients.
- Early recognition and multidisciplinary management improve the chances of disease control.

## Data Availability

The data utilized in the development of this manuscript are available upon request from the corresponding author.

## References

[1] Shidnia H, Hornback NB, Lingeman R, Barlow P. Extranodal lymphoma of the head and neck area. Am J Clin Oncol. 1985;8(3):235. 10.1097/00000421-198506000-00008 4050742

[2] Sekhon S, Kolte S, Gupta K, Yadav A. Atypical clinical presentations of lymphomas: two case reports. J Mahatma Gandhi Inst Med Sci. 2016;21(1):50.

[3] Delecluse HJ, Anagnostopoulos I, Dallenbach F, Hummel M, Marafioti T, Schneider U, *et al*. Plasmablastic lymphomas of the oral cavity: a new entity associated with the human immunodeficiency virus infection. Blood. 1997;89(4):1413–20. 9028965

[4] Bhatt R, Naha K, Desai DS. Plasmablastic Lymphoma. In: StatPearls. Treasure Island: StatPearls Publishing; 2025. 30422570

[5] Bailly J, Jenkins N, Chetty D, Mohamed Z, Verburgh ER, Opie JJ. Plasmablastic lymphoma: an update. Int J Lab Hematol. 2022;44(Suppl 1(Suppl 1)):54–63. 10.1111/ijlh.13863 PMC9545967 36074710

[6] Harmon CM, Smith LB. Plasmablastic lymphoma: a review of clinicopathologic features and differential diagnosis. Arch Pathol Lab Med. 2016;140(10):1074–8. 10.5858/arpa.2016-0232-RA 27684979

[7] Li JW, Peng HL, Zhou XY, Wang JJ. Plasmablastic lymphoma: current knowledge and future directions. Front Immunol. 2024;15: 1354604. 10.3389/fimmu.2024.1354604 PMC10896986 38415257

[8] Loghavi S, Alayed K, Aladily TN, Zuo Z, Ng SB, Tang G, *et al*. Stage, age, and EBV status impact outcomes of plasmablastic lymphoma patients: a clinicopathologic analysis of 61 patients. J Hematol Oncol. 2015;8: 65. 10.1186/s13045-015-0163-z PMC4472407 26055271

[9] Sharma D, Khurana N, Narula V, mohanty S. Plasmablastic lymphoma presenting as gingival growth in a HIV positive patient: a case report. J Oral Maxillofac Surg Med Pathol. 2016;28(4):366–9.

[10] Horwitz SM, Zelenetz AD, Gordon LI, Wierda WG, Abramson JS, Advani RH, *et al*. NCCN guidelines insights: non-Hodgkin’s lymphomas, version 3.2016. J Natl Compr Canc Netw. 2016;14(9):1067–79. 10.6004/jnccn.2016.0117 27587620

